# Bupivacaine modulates the apoptosis and ferroptosis in bladder cancer via phosphatidylinositol 3-kinase (PI3K)/AKT pathway

**DOI:** 10.1080/21655979.2022.2036909

**Published:** 2022-03-04

**Authors:** Jianli Hao, Weiqing Zhang, Zeqing Huang

**Affiliations:** Department of Anesthesiology, Cancer Hospital of China Medical University, Liaoning Cancer Hospital & Institute, Shenyang, China

**Keywords:** Bupivacaine, bladder cancer, apoptosis, ferroptosis, PI3K/AKT signaling pathway

## Abstract

The study aimed to explore the effects of local anesthetic bupivacaine on bladder cancer cells in *vivo* and in *vitro*. The cytotoxicity was detected by MTT assay. Apoptosis was measured by Hoechst 33342 staining and TUNEL. The contents of Fe^2+^, Malondialdehyde (MDA), Glutathione (GSH) and reactive oxygen species (ROS) were evaluated by the corresponding kit. Mitochondrial membrane potential was assessed by JC-1 kit. HE staining, TUNEL and immunohistochemistry were used to detect the xenografted tumors. Protein expression was estimated by Western blot. Bupivacaine significantly inhibited the activity of T24 cells and 5637 cells at 0.25–16 mM. Bupivacaine promoted cell apoptosis with increased concentration. bupivacaine inhibited the expression of Bcl-2 and increased the expression of Bax and cytochrome C. Moreover, bupivacaine amplified the level of Fe^2+^ and ROS, and restrained the expression of cystine/glutamic acid reverse transporter (xCT) and glutathione peroxidase 4 (GPX4). Further results showed that bupivacaine decreased mitochondrial membrane potential, reduced GSH, and increased MDA levels. Besides, bupivacaine attenuated the phosphorylation of PI3K, Akt, and mTOR. In addition, bupivacaine suppressed the growth of xenografted tumors, induced apoptosis and ferroptosis, and inhibited the activity of PI3K/AKT signaling pathway in xenografted tumors. Bupivacaine could induce apoptosis and ferroptosis by inhibiting PI3K/Akt signaling pathway in bladder cancer cells.

## Graphical abstract

**Figure:**
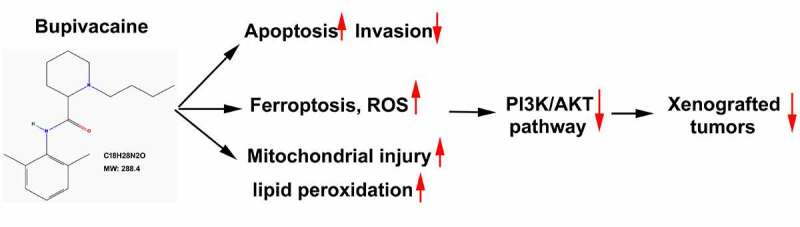

## Introduction

Bladder cancer is one of the most common malignant tumors in the world [1]. Up to 20% of the patients were diagnosed as invasive bladder cancer (T2-T4 stage) [2]. In the treatment of invasive bladder cancer, besides conventional cystectomy, pelvic lymph node dissection should be performed at the same time [3]. Moreover, systemic treatment should also be carried out before and after operation, such as chemotherapy [3]. However, surgery itself could cause significant stress reaction, which directly or indirectly promote the growth of tumor [4]. On the other hand, surgical trauma has obvious cellular immunosuppressive effect, which hinders the clearance of residual tumor cells [5,6]. Therefore, the risk of tumor metastasis and recurrence in perioperative patients is great [5,7,8]. In recent years, a large number of clinical retrospective studies have shown that the application of local anesthetics could prevent postoperative recurrence of cancer patients [9,10]. It has been reported that propofol could inhibit cell activity and promote cell apoptosis through inhibiting the mTOR/P70S6K signaling pathway mediated by HOTAIR [11]. It was found that lidocaine could directly and effectively inhibit the proliferation and migration of lung cancer cells in vitro [12]. Therefore, local anesthetics might possess the function of killing or inhibiting tumor cells.

Bupivacaine, as one of the commonly used local anesthetics, is used in local infiltration, epidural and intrathecal anesthesia through its sodium channel block characteristics [13,14]. Studies have found that bupivacaine could inhibit the growth of tumor cells [15]. Zheng et al. found that bupivacaine-induced apoptosis and necrosis by consuming nicotinamide adenine dinucleotide (NAD^+^) in neuroblastoma cells, and exogenous NAD^+^ reversed the process of cell death [16]. Moreover, Bupivacaine could enhance apoptosis of ovarian and prostate cancer cells and reduce the occurrence of gastric cancer [15,17]. However, the effect of bupivacaine on bladder cancer cells has not yet been reported.

Ferroptosis is a non-apoptotic cell death mode driven by lipid peroxidation [18]. In morphology, the main manifestations of ferroptosis were the decrease of mitochondrial volume, the increase of double membrane density and the decrease or disappearance of mitochondrial cristae [19]. In biochemical aspect, glutathione was exhausted, GPX4 activity was decreased, lipid oxide metabolism was blocked, and then divalent iron ions oxidized lipid to produce a large number of ROS, which promoted ferroptosis of cells [18,20]. Studies have shown that many drugs could inhibit the growth of tumor cells by inducing ferroptosis [21,22]. Sorafenib could increase the level of lipid oxidation in hepatocytes and induce ferroptosis [23]. Wang et al. found that Pseudolaric acid B activated NADPH Oxidase 4 in glioma cells, inhibited the activity of xCT on cell membrane, and promoted ferroptosis of glioma cells [24]. However, the relationship between anesthetic-mediated inhibition of tumor cell and ferroptosis still needs to be further confirmed.

In this study, we hypothesized that bupivacaine could inhibit the growth of bladder cancer cell. We aimed to study the effects of bupivacaine on bladder cancer apoptosis, ferroptosis and oxidative stress in *vitro* and *vivo*. Moreover, its underlying mechanism was further explored in bladder cancer cells, especially the role of PI3K/AKT/mTOR.

## Materials and methods

### Cell culture

Human bladder cancer cells (T24 cells and 5637 cells) and human uroepithelial cells (sv-huc-1 cells) were obtained from the American type culture collection (ATCC, USA). Cells were maintained in Dulbecco’s Modified Eagle Medium (DMEM, Gibco) containing 10% fetal bovine serum (FBS, Gibco) and cultured in an incubator of 5% CO_2_ at 37°C.

### MTT assay

T24 cells and 5637 cells were seeded into 96 well plates according to the concentration of 5x10^3^/well. After 24 h of culture, T24 cells were treated for another 24 h with different doses of bupivacaine (0, 0.01, 0.025, 0.05, 0.1, 0.25, 0.5, 1, 2, 4, 8, or 16 mM). 20 μl MTT (5 mg/ml, Sigma-Aldrich, USA) reagent was added to each well (200 μl) and incubated for 4 h in 5% CO_2_ incubator at 37°C. Following that, the supernatant was discarded, and 200 μl dimethyl sulfoxide (DMSO, Sigma-Aldrich, USA) was added to each well. After shaking for 30 s, the absorbance of each group was detected at 570 nm by a microplate reader (BioTek Instruments, USA).

### Hoechst 33342 staining

T24 cells and 5637 cells were seeded into 96 well plates according to the concentration of 5x10^3^/well. After 24 h of culture, T24 cells were treated for another 24 h with bupivacaine (0, 0.25, 0.5, 1 mM) at 37°C. After fixed for 10 min with 37% formaldehyde at room temperature, the cells were stained for 5 min with 2 μg/mL Hoechst 33342 at room temperature in dark. The nuclear morphology of the cells was examined by fluorescence microscopy (magnification: 400× objective; Nikon C2 Plus, Tokyo, Japan).

### Flow cytometry

The of apoptosis was measured by flow cytometry. Annexin V/PI double staining was performed using an apoptosis detection kit (Beyotime) according to the manufacturer’s instructions. Briefly, following exposure to various treatments, cells were washed with ice cold PBS and binding buffer, suspended in 300 µL binding buffer containing annexin V and PI, and then incubated for 5 min at room temperature in dark. The cell apoptosis was detected within 1 h using flow cytometry.

### TUNEL assay

The effect of bupivacaine on T24 cells and 5637 cells apoptosis was measured by TUNEL Detection Kit (Roche, Basel, Switzerland). Briefly, cells treated with bupivacaine (0, 0.25, 0.5, or 1 mM) were collected, and which were suspended with PBS. Cells were fixed for 25 min with 4% paraformaldehyde at 4°C, and then incubated for 5 min at room temperature with Proteinase K. Following that, cells were transferred into 4-well chamber slide, and then 50 μl of TUNEL reaction mixture was added to each well. Next, cells were incubated in dark for 1 h, and stained nucleus with 1 μg/ml DAPI (Solarbio Life Science, China) at 37°C. Five different fields of vision of the same section were observed by fluorescence microscope (magnification: 400 × objective; Nikon C2 Plus, Tokyo, Japan), and the average value was taken to record the apoptosis.

For animal, the pathological sections were detected by TUNEL Detection Kit (Beyotime) according to the instructions. The staining results were photographed using a microscope (magnification: 200× objective; Olympus, Tokyo, Japan), and the positive apoptotic cells were brown nuclei, and then the results were analyzed using Image J 1.48 v software (NIH).

### Transwell assay

To estimate whether bupivacaine inhibited invasion of T24 cells and 5637 cells, matrigel invasion test was used. 10^4^ cells/well were seeded in 200 μL of FBS-free medium pre-coated with matrigel in the upper chamber. Fill the lower well with 1 mL of DMEM medium (with 10% FBS) to attract cell invasion. After incubation for 24 h, the cells on the surface of the membrane were fixed with 4% paraformaldehyde, stained with crystal violet, and observed under a microscope. A total of five random fields were taken for each filter, and the number of cells was directly counted.

### Determination of Fe2^+^ concentration

Cells treated with bupivacaine (0, 0.25, 0.5, or 1 mM) were collected and lysed with RIPA lysate (Beyotime). The supernatant was collected by centrifugation (12,000 × rpm, 10 min). 50 μl supernatant/well from each group and 50 μl/well of 0.1 M hydrochloric acid were mixed in 96-well plate, and incubated for 30 min at 25°C. Following that, 100 μl iron probe was added into each well, and cells were incubate for 60 min at 25°C. The absorbance was detected at 562 nm with a microplate reader (BioTek Instruments, USA).

### Detection of oxidative stress index

As previous study [25], Glutathione (GSH) and malondialdehyde (MDA) were detected using the ELISA kit provided by Nanjing Jiancheng Biotechnology Co., Ltd. The test was performed according to the instructions.

### Determination of intracellular ROS

The cells were seeded on a 6-well plate (2 × 10^5^ cells/well) and cultured for 24 h at 37°C. The cells were treated with bupivacaine (0, 0.25, 0.5, or 1 mM) was added and were cultured for 24 h in a 5% CO_2_ incubator at 37°C. Then, the culture medium was discarded, and the cells were incubated with DCFH-DA (50 μm/well) in the incubator for 30 min. The supernatant was discarded and the cells were washed by PBS for three times. The fluorescence intensity was detected by fluorescence microscope (magnification: 200 × objective; Nikon C2 Plus, Tokyo, Japan). The excitation wavelength and detection wavelength were 488 nm and 525 nm, respectively.

### Detection of mitochondrial membrane potential (MMP)

MMP was measured by JC-1 method as previous study [26]. Briefly, the cells were seeded on 6-well plates (2 × 10^5^ cells/well) and cultured overnight at 37°C. After discarding the supernatant, the cells were treated with bupivacaine (0, 0.25, 0.5, or 1 mM) and cultured for 24 h in a 5% CO_2_ incubator at 37°C. Following that, cells were mixed with JC-1 staining working solution, and further cultured at 37°C for 30 min, and then washed with PBS for 3 times. Fluorescence (excitation: 488 nm, emission: 525 nm) was measured by flow cytometry (BD Bioscience, USA).

### Western blot

T24 and 5637 cells were treated with RIPA reagents (Beyotime), and then the total protein was obtained by centrifugation (12,000 × rpm, 10 min). The protein was quantified with BCA protein assay kit (beyotime). 50 μg protein/lane was isolated in 10% SDS-PAGE, and then transferred to PVDF membrane. The membranes were sealed for 2 h at room temperature with 5% skimmed milk, and then incubated with primary antibody [xCT (1:1000, ab175186, Abcam); GPX4 (1:1000, ab40993, Abcam); Bcl-2 (1:1000, ab117115, Abcam); Bax (1:1000, ab3191, Abcam); Cytochrome C (1:1000, ab13354, Abcam); PI3K (1:1000, #4292, Cell Signaling Technology); p-PI3K p85α (1:1000, #17,366, Cell Signaling Technology); AKT (1:1000, #9272, Cell Signaling Technology); p-AKT Thr308 (1:1000, #13,038, Cell Signaling Technology); p-AKT Ser473 (1:1000, #4060, Cell Signaling Technology); mTOR (1:1000, #2972, Cell Signaling Technology); p-mTOR Ser2448 (1:1000, #5536, Cell Signaling Technology); β-actin (1:1000, ab8224, Abcam)] overnight at 4°C. Then the membranes were incubated for 2 h at room temperature with second antibody [Goat Anti-Rabbit IgG H&L (1:5000, ab96899, Abcam) or Goat Anti-Mouse IgG H&L (1:5000, ab96879, Abcam)]. The bands were visualized with chemiluminescent reagents (Beyotime) and photographed on a gel image analysis system (Bio-Rad, USA).

### Establishment of xenografted tumors

A mouse model of bladder cancer was constructed by inoculating T24 cells were injected into the axilla of mice to construct a xenografted tumors of bladder cancer. After 7 days of injection, 16 mice with 6–8 mm diameter visible tumor were randomly divided into two groups: control group (n = 8) and bupivacaine treatment group (n = 8). Following that, bupivacaine (11.5 mg/kg) was intraperitoneally injected once a day. The tumor volume and weight were recorded every 3 days after the administration. 27 days later, nude mice were euthanized, and tumor tissue was isolated and photographed. The animal study was performed according to the Guide for the Care and Use of Laboratory Animals of Cancer Hospital of China Medical University, and approved by the Ethical Commission of Cancer Hospital of China Medical University.

### Hematoxylin & eosin (HE) staining

The tumor tissue was fixed in 10% formalin, embedded in paraffin and made into 4.5 μm sections. Then, the sections were stained with HE dyeing solution and observed under a microscope (magnification: 200× objective; Olympus, Tokyo, Japan).

### Immunohistochemistry

The sections were dewaxed, antigen-repaired, and blocked, and then they were incubated with primary antibody xCT and GPX4 overnight at 4°C. Next, the sections were treated with secondary antibody for 2 h at room temperature. Following that, the sections were stained with DAB and then re-stained with hematoxylin. Brown yellow represented positive cells and blue represented nucleus. The images were obtained under a microscope (magnification: 400× objective; Olympus, Tokyo, Japan) and analyzed by Image J 1.48 v software (NIH).

### Statistical analysis

The experimental data were analyzed by SPSS 20.0, and the data were represented using means ± SD. Differences between groups were analyzed by t-test (two groups) or one-way ANOVA (multiple groups). P < 0.05 was statistically significant.

## Results

### Bupivacaine induced apoptosis of bladder cancer cells

**Figure 1.**
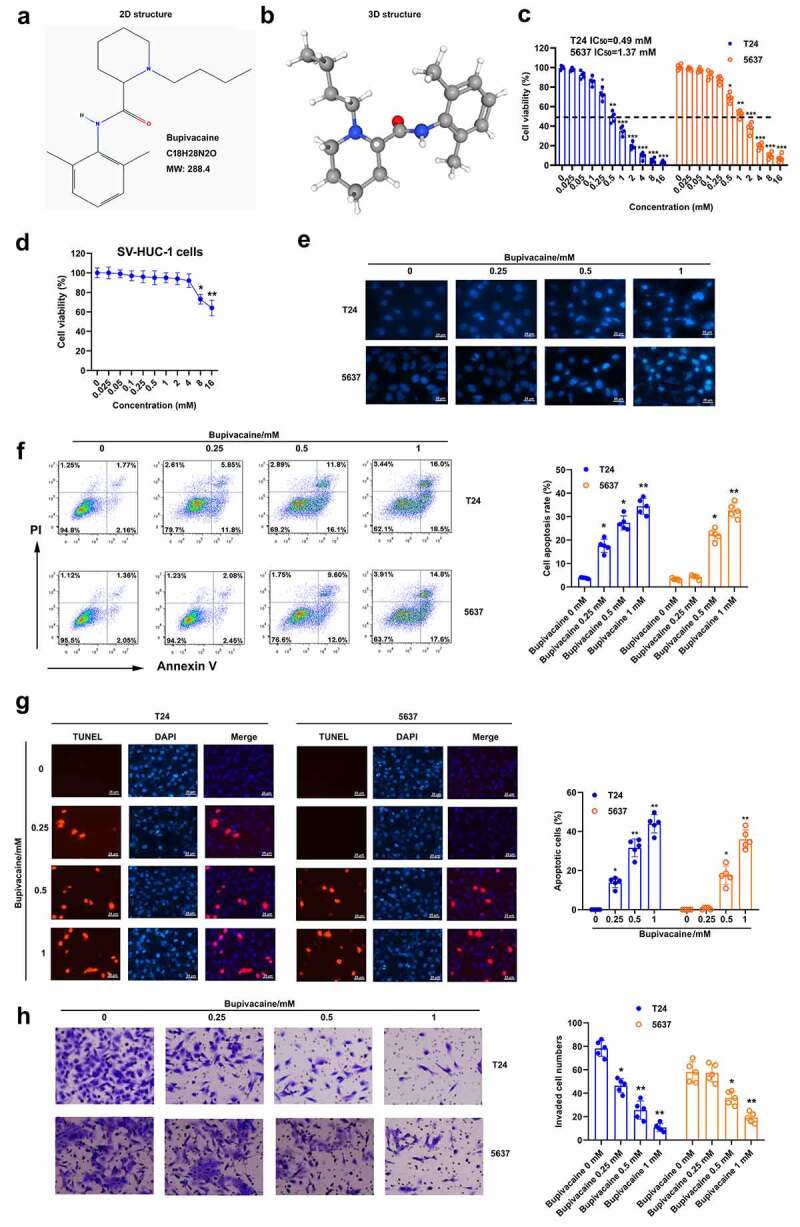
Bupivacaine promoted bladder cancer cell apoptosis. (a) and (b) 2D and 3D chemical structure of bupivacaine were obtained through PubChem database (https://pubchem.ncbi.nlm.nih.gov/compound/12313549). (c) and (d) Bladder cancer cell (T24 cells and 5637 cells) and human uroepithelial cells (sv-huc-1 cells) were treated with 24 h with bupivacaine (0, 0.01, 0.025, 0.05, 0.1, 0.25, 0.5, 1, 2, 4, 8, or 16 mM), and cell viability was detected by MTT assay. (e) Bladder cancer cells were treated for 24 h with bupivacaine (0, 0.25, 0.5. 1 mM), and nuclear morphology was measured by Hoechst 33,342 staining. (f) and (g) Apoptosis was tested by flow cytometry and TUNEL assay. (h) the invasion of bladder cancer cells was evaluated through transwell assay. *P < 0.05 vs. the group treated without bupivacaine, **P < 0.01 vs. the group treated without bupivacaine, ***P < 0.001 vs. the group treated without bupivacaine.

The effect of bupivacaine on the growth of bladder cancer cells was explored. The 2D and 3D chemical structures of bupivacaine were obtained from PubChem database (https://pubchem.ncbi.nlm.nih.gov/compound/12313549) (Figure 1(a,b)). The cytotoxicity of bupivacaine on T24 cells and 5637 cells was detected. The results showed that bupivacaine had no effect on the activity of T24 cells and 5637 cells in the concentration range of 0–0.1 mM, but had obvious inhibitory effect on the activity of T24 cells and 5637 cells at 0.25 mM and 0.5 mM, respectively (Figure 1(c)). It was found that the activity of sv-huc-1 cells was reduced only when the concentration of bupivacaine was greater than or equal to 8 mM (Figure 1(d)). It was demonstrated that the four doses selected in this paper (0, 0.25, 0.5 and 1 mM) have no effect on normal cells, but only on cancer cells. Therefore, 0, 0.25, 0.5 and 1 mM were used in the following experimental study. Moreover, results of Hoechst 33,342 staining, flow cytometry and TUNEL showed that bupivacaine could induce the apoptosis of T24 and 5637 cells at 0.25, 0.5 and 1 mM (Figure 1(e–g)). The number of cells that invaded the lower chamber was significantly decreased by the treatment of bupivacaine with a concentration dependent (Figure 1(h). These results suggested that bupivacaine (0.25, 0.5, 1 mM) restrained cell growth and invasion in bladder cancer cells.

### Bupivacaine induced ferroptosis in bladder cancer cells

**Figure 2.**
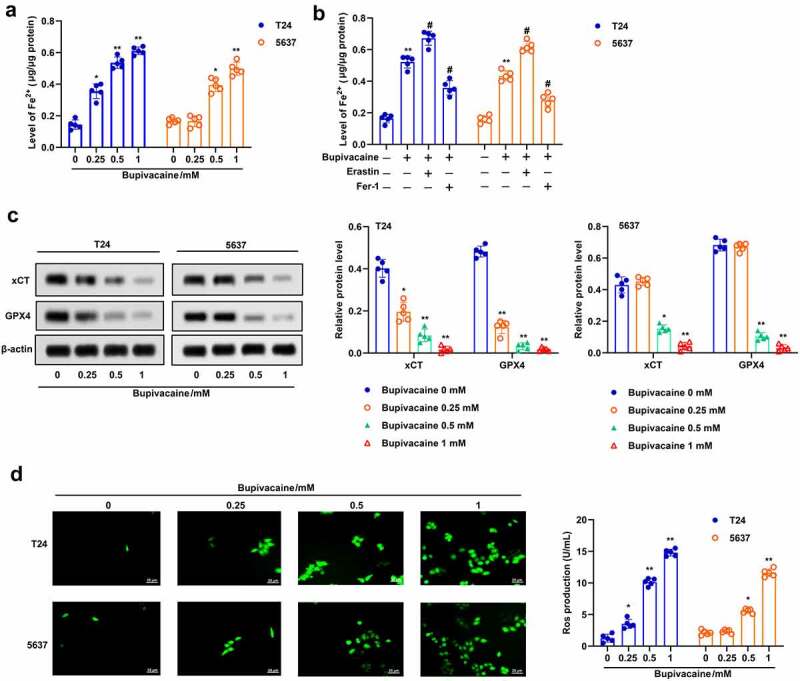
Bupivacaine promoted ferroptosis in bladder cancer cells. T24 cells and 5637 cells were treated for 24 h with bupivacaine (0, 0.25, 0.5, 1 mM). (a) and (b) The concentration of Fe^2+^ was detected. (c) The protein expression of xCT and GPX4 were measured by Western blot. (d) The level of ROS was detected by DCFH-DA probe. *P < 0.05 vs. the group treated without bupivacaine, **P < 0.01 vs. The group treated without bupivacaine, ^#^P < 0.05 vs. the group treated with bupivacaine.

In order to further study the effect of bupivacaine on bladder cancer cells, we detected whether bupivacaine could induce ferroptosis in bladder cancer cells. First, our finding showed that bupivacaine increased Fe^2+^ concentration in T24 and 5637 cells in a concentration-dependent manner (Figure 2(a)). Moreover, erasin (ferroptosis activator) enhanced bupivacaine-induced upregulation of Fe^2+^ concentration in T24 and 5637 cells, while Fer-1 (ferroptosis inhibitor) reversed the effect of bupivacaine on Fe^2+^ concentration (Figure 2(b)). In addition, our results showed that bupivacaine inhibited the protein expression of xCT and GPX4 in T24 and 5637 cells in a concentration-dependent manner (Figure 2(c)). In addition, bupivacaine increased ROS levels in T24 and 5637 cells with increasing concentration (Figure 2(d)). These data indicated that bupivacaine promoted ferroptosis in bladder cancer cells.

### Bupivacaine enhanced oxidative stress in bladder cancer cells

**Figure 3.**
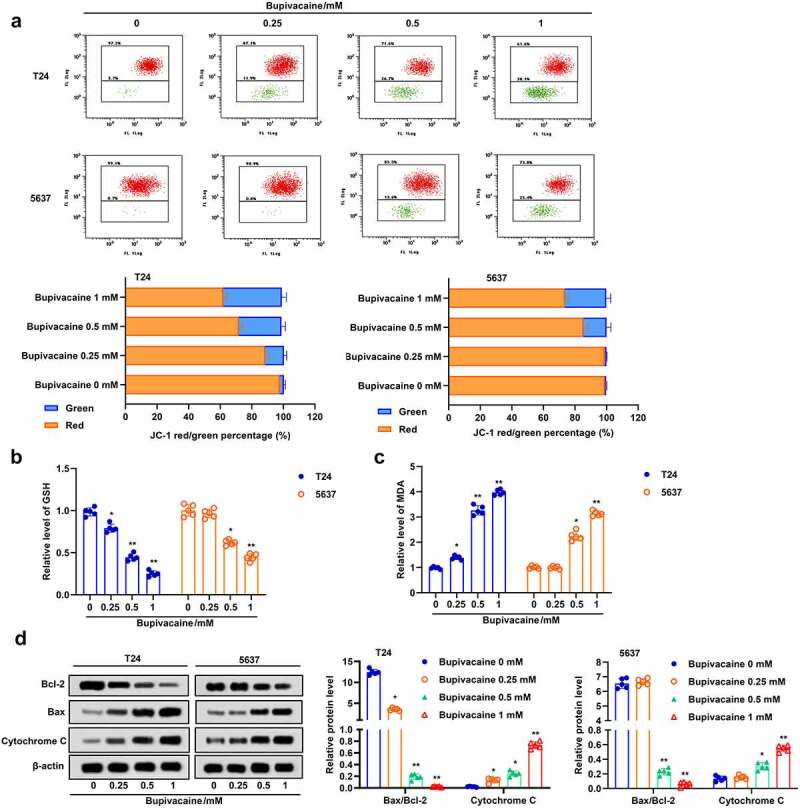
Bupivacaine promoted mitochondrial injury and lipid peroxidation. The cells were treated for 24 h with bupivacaine (0, 0.25, 0.5, 1 mM). (a) Mitochondrial membrane potential was detected by JC-1 staining. (b) and (c) GSH and MDA levels were measured by ELISA. (d) The expression of Bcl-2, Bax and cytochrome C were detected by Western blot. *P < 0.05 vs. the group treated without bupivacaine, **P < 0.01 vs. The group treated without bupivacaine.

Apoptosis and ferroptosis are usually accompanied by increasing oxidative stress [27]. Here, we examined the effect of bupivacaine on oxidative stress in T24 and 5637 cells. The results suggested that bupivacaine increased the proportion of JC-1 stained green, but decreased the proportion of JC-1 stained red, which indicated that bupivacaine decreased the mitochondrial membrane potential in T24 and 5637 cells (Figure 3(a)). Besides, the GSH level was decreased and the MDA level was increased with the increase of bupivacaine concentration (Figure 3(b,c)). Furthermore, the results indicated that bupivacaine inhibited the expression of Bcl-2 and promoted the expression of Bax and cytochrome C in T24 and 5637 cells (Figure 3(d)). These results showed that Bupivacaine increased oxidative stress, and promoted mitochondrial apoptosis in bladder cancer cells.

### Bupivacaine increased apoptosis and ferroptosis in bladder cancer cells by inhibiting PI3K/Akt signaling pathway

**Figure 4.**
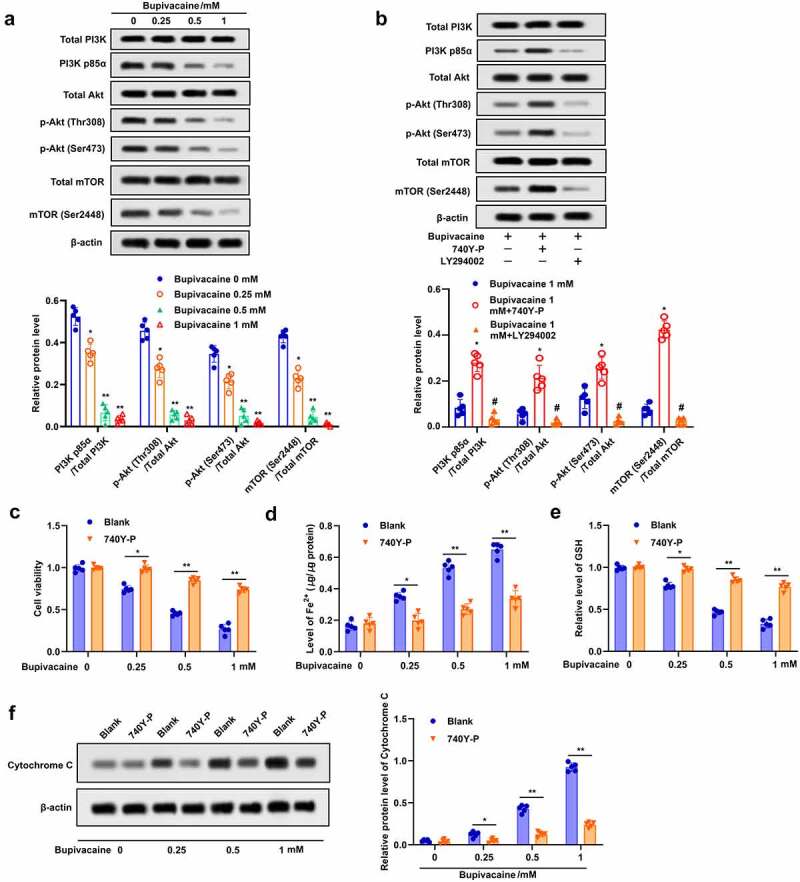
Bupivacaine induced apoptosis and ferroptosis by inhibiting PI3K/AKT signaling pathway. (a) The cells treated for 24 h with bupivacaine (0, 0.25, 0.5, 1 mM), and then the expression of PI3K/AKT signaling pathway related proteins were detected by Western blot. (b) The cells were treated with bupivacaine and 740Y-P (or LY294002), the expression of PI3K/AKT signaling pathway related proteins were detected by Western blot. The cells were treated with bupivacaine alone or with bupivacaine and 740Y-P. (c) Cell viability was measured by MTT assay. (d) The concentration of Fe^2+^ was detected. (e) GSH level was detected by ELISA. (f) The expression of cytochrome C protein was detected by Western blot. *P < 0.05 vs. the group treated without bupivacaine, **P < 0.01 vs. The group treated without bupivacaine, ^#^P < 0.05 vs. the group treated with bupivacaine.

In order to further study the underlying mechanism of bupivacaine-induced apoptosis and ferroptosis, the effect of bupivacaine on PI3K/Akt signaling pathway was evaluated. The results indicated that bupivacaine reduced the phosphorylation of PI3K, p58a, Akt (thr308), Akt (ser473), mTOR (ser2448) in T24 cells (Figure 4(a)). Bupivacaine-induced inhibition of PI3K/Akt signaling pathway was attenuated by 740Y-P and enhanced by LY294002 (Figure 4(b)). In addition, 740Y-P could effectively restrained bupivacaine-induced inhibition of cell activity and GSH, and weaken bupivacaine-induced increase of Fe^2+^ concentration (Figure 4(c–e)). In addition, 740Y-P reversed the upregulated effect of bupivacaine on cytochrome C expression in T24 cells (Figure 4(f)). Taken together, these results indicated that bupivacaine enhanced apoptosis and ferroptosis in bladder cancer cells through inhibiting PI3K/Akt signaling pathway.

### *Bupivacaine inhibited the growth of xenografted tumor of bladder cancer in* vivo

**Figure 5.**
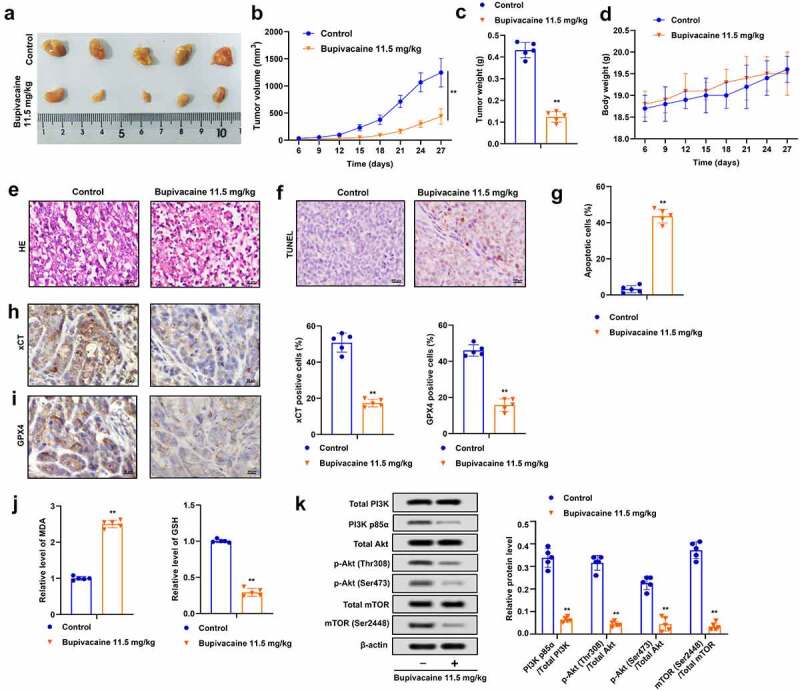
Bupivacaine inhibited the growth of xenografted tumors. Xenografted tumors were treated with or without bupivacaine. (a) and (b) Tumor size was measured every three days. (c) The weight of the tumors was detected. (d) The weight of nude mice was tested. (e) Pathological changes were detected by HE staining. (f) and (g) Apoptosis was detected by TUNEL. (h) and (i) xCT and GPX4 expression were detected by immunohistochemistry. (j) MDA and GSH levels were detected by ELISA. (k) The expression of PI3K/AKT signaling pathway related proteins were detected by Western blot. **P < 0.01 vs. control group.

It was further assessed whether bupivacaine restrained the growth of bladder cancer cell in *vivo*. The results indicated that bupivacaine significantly inhibited the volume and weight of xenografted tumors (Figure 5(a–c)), and bupivacaine had no effect on the weight of mice compared with the control group (Figure 5(d)). Moreover, bupivacaine reversed the pathological changes of tumor (Figure 5(e)), and bupivacaine treatment group showed more apoptosis than the control group (Figure 5(f,g)). Besides, compared with the control group, the expression of xCT and GPX4 and GSH level were decreased, while MDA level was increased in the bupivacaine treatment group (Figure 5(h–j)). In addition, bupivacaine suppressed the phosphorylation of PI3K, p58α, Akt (thr308), Akt (ser473), mTOR (ser2448) in xenografts (Figure 5(k)), which was consistent with the results of cell experiment. These results indicated that bupivacaine reduced the growth of xenografted tumor of bladder cancer.

## Discussion

Local anesthetics have a wide range of effects. More and more evidence showed that local anesthetics have inhibitory effects on a variety of tumor cells [28–32]. Bupivacaine could induce ovarian cancer and prostate cancer cell death in a certain concentration range, and inhibit the proliferation of colon cancer and pancreatic cancer cells [17]. Bupivacaine can significantly reduce the number of Ki67 positive Caco-2 cells and inhibit the proliferation of colon cancer cells [33]. In this paper, the results showed that the proliferation activity of T24 cells and 5637 cells treated with bupivacaine (0.25–16 mM) was significantly inhibited, and our study also confirmed that bupivacaine promoted apoptosis and ferroptosis in bladder cancer cells by inhibiting PI3K/AKT/mTOR signaling pathway.

Inducing apoptosis is one of the main methods to inhibit the growth of tumor cells [34]. It was found that the number of apoptotic cells in all tumor groups were increased with the raise of bupivacaine concentration and exposure time [30]. Chang et al. found that bupivacaine could effectively induce apoptosis of human breast cancer cells in vitro [35]. Similarly, treatment of human thyroid cancer cells with bupivacaine resulted in inhibition of cell viability and clonal formation, and induced apoptosis through mitochondrial damage and mitogen activated protein kinase (MAPK) activation [36]. In this paper, the results showed that bupivacaine could promote cell apoptosis, decrease the expression of anti-apoptotic protein Bcl-2, and increased the expression of pro-apoptotic protein Bax and cytochrome C in T24 cells and 5637 cells.

Ferroptosis is regulated by many pathways and genes, including iron homeostasis and lipid peroxidation [37]. When the iron balance is disturbed, excessive free iron could increase the production of ROS through fenton method, thus promote the lipid peroxidation to induce cell death [38]. xCT is a functional subunit of system xc- (glutamic acid/cystine transporter). The inhibition of xCT can promote the accumulation of intracellular iron and induce ferroptosis [39]. Glutathione peroxidase-4 (GPX4) is one of the important markers of ferroptosis [40]. Erastin could reduce the activity of GPX4 by inhibiting the synthesis of glutathione, resulting in the block of lipid peroxidation metabolism, thus inducing cell death [41,42]. Mitochondrial membrane potential (MMP) plays an important role in mitochondrial apoptosis. At the same time, ferroptosis could induce the change of mitochondrial membrane structure, resulting in downregulation of MMP [43]. Ruscogenin enhanced the expression of transferrin and iron transporter, thus increased the concentration of Fe^2+^ in cells, which induce ferroptosis in pancreatic cancer cells [44]. Solasonine can increase ROS level, inhibit the expression of GPX4 and GSS, and promote ferroptosis of hepatoma cells. Moreover, ferroptosis inhibitor can reverse the effect of solasonine on hepatoma cells [45]. GSH is an intracellular antioxidant, and MDA is an indicator of lipid peroxidation, which plays important roles in cell ferroptosis [46]. It was found that bupivacaine induced oxidative damage in SH-SY5Y cells by reducing GSH content and increasing MDA content [47]. We speculated that bupivacaine could induce ferroptosis in bladder cancer cells. Interestingly, our results showed that bupivacaine-induced ferroptosis, increased Fe^2+^ concentration of T24 cells, reduced the expression of xCT and GSX-4, GSH level, and MMP, and increased ROS and MDA levels. Moreover, bupivacaine decreased mitochondrial membrane potential and increased cytochrome C expression, indicating that bupivacaine caused to mitochondrial dysfunction. However, whether bupivacaine alter the mitochondrial morphology of the tumor cells and thus affect their energy metabolism need to be further explored in our future study.

PI3K/AKT signaling pathway is considered to be a regulatory factor of cell metabolism, which regulates cell function by sensing the metabolic state in cells, including apoptosis and ferroptosis [48–50]. Levobupivacaine inhibited breast cancer cell proliferation and induced apoptosis through inhibition of PI3K/AKT/mTOR signaling pathway [51]. Bupivacaine could induce apoptosis of neuroblastoma cells by inhibiting the phosphorylation level of PI3K [52]. Bupivacaine, a common local anesthetic, could induced apoptosis and PI3K/PKB pathway inactivation [47]. Bupivacaine inhibits proliferation and metastasis of hepatocellular carcinoma cells via suppressing PI3K/Akt and MAPK signaling [53]. PI3K/AKT/mTOR signaling pathway promotes ferroptosis and oxidative stress through regulating SREBP1 mediated lipogenesis, and PI3K inhibitor could block the effect [49]. In this paper, our results indicated that bupivacaine inhibited the phosphorylation of PI3K, Akt, mTOR. Further verification demonstrated that PI3K inhibitor could reverse the effects of bupivacaine on T24 cell activity, Fe^2+^ concentration, GSH level, and cytochrome C expression. Finally, our findings suggested that bupivacaine inhibited the growth of xenografted tumors and promoted cell apoptosis and ferroptosis in vivo.

## Conclusion

Our results showed a new insight into the function of bupivacaine in cancer treatment. Moreover, it was firstly demonstrated that bupivacaine could promote ferroptosis and apoptosis in bladder cancer cells by inhibiting PI3K/AKT signaling pathway. Our data provided experimental evidence for the clinical application of bupivacaine in treatment of bladder cancer.

## Data Availability

The datasets used and/or analyzed during the current study are available from the corresponding author on reasonable request.

## Highlights

- Bupivacaine inhibited growth of bladder cancer cell in vitro and vivo.
- Bupivacaine induced ferroptosis through promoting the increase of Fe^2+^ and ROS and reducing the expression of xCT and GPX4.
- Inactivity of PI3K/AKT signaling pathway might be the main molecular mechanism of bupivacaine against bladder cancer.
